# Regulatory Guidelines for the Analysis of Therapeutic Peptides and Proteins

**DOI:** 10.1002/psc.70001

**Published:** 2025-02-08

**Authors:** Yomnah Y. Elsayed, Toni Kühl, Diana Imhof

**Affiliations:** ^1^ Pharmaceutical Biochemistry and Bioanalytics, Pharmaceutical Institute University of Bonn Bonn Germany; ^2^ Department of Pharmaceutical Analytical Chemistry, Faculty of Pharmacy Ain Shams University Cairo Egypt

**Keywords:** bioanalytics, excipients, formulation, guidelines, peptides, proteins

## Abstract

Peptides and proteins have become increasingly important in the treatment of various diseases, including infections, metabolic disorders, and cancers. Over the past decades, the number of approved peptide‐ and protein‐based drugs has grown significantly, now accounting for about 25% of the global pharmaceutical market. This increase has been recorded since the introduction of the first therapeutic peptide, insulin, in 1921. Therapeutic peptides and proteins offer several advantages over small molecule drugs, including high specificity, potency, and safety; however, they also face challenges related to instability in liquid formulations. To address this issue, numerous formulation techniques have been developed to enhance their stability. In either state, physical and chemical characterization of the peptide or protein of interest is crucial for ensuring the identity, purity, and activity of these therapeutic agents. Regulatory bodies such as the FDA, ICH, and EMA have established guidelines for the analysis, stability testing, and quality control of peptides and biologics to ensure the safety and effectiveness of these drugs. In the present review, these guidelines and the consequences thereof are summarized and provided to support the notion of developing tailored bioanalytical workflows for each peptide or protein drug.

## Graphical abstract

Guidelines from regulatory agencies support the analysis and quality control of peptide and protein drugs. Key aspects of the analysis process are summarized here.

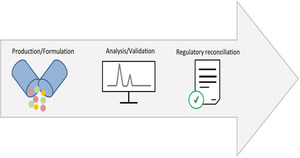

## 1 Introduction

**FIGURE 1:**
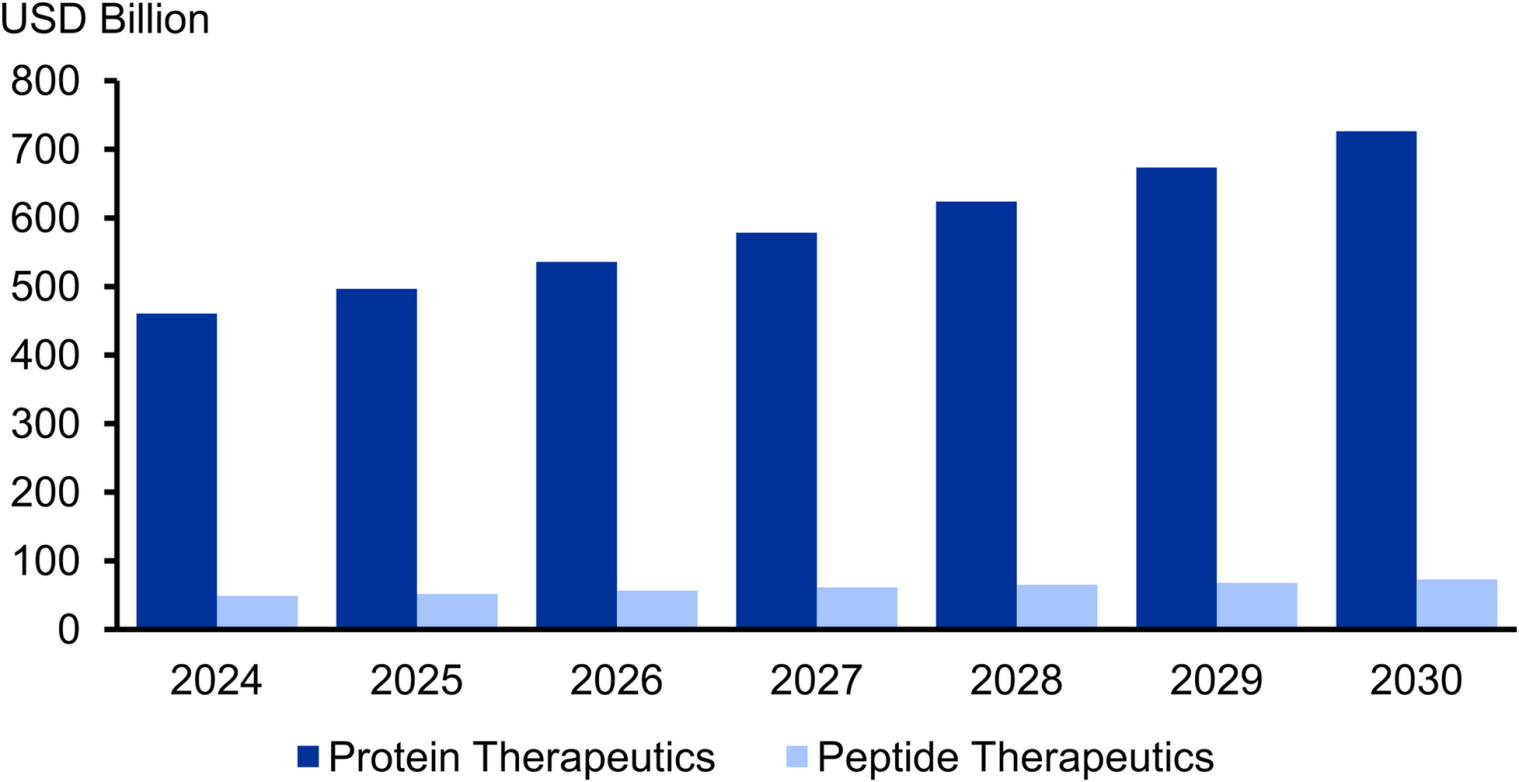
Expected peptide and protein therapeutics market development until 2030 [1–9].

The therapeutic significance of peptides and proteins has increased dramatically over the past decades, especially in the treatment and management of various pathophysiological conditions, including bacterial and viral infections, metabolic and endocrine disorders, and various types of cancer [1]. As a consequence, the number of approved peptide and protein drugs has risen sharply and now accounts for approximately 25% of the pharmaceutical market, representing sales of more than USD 500 billion (peptides: > USD 40 billion, biologicals: > USD 460 billion) (Figure 1) [2, 3, 4, 5, 6, 7, 8, 9]. The introduction of biomolecules as therapeutic agents dates back to the isolation of the first therapeutic peptide, namely, insulin, in 1921 [4, 10, 11]. Since then, peptides and biologics (i.e., protein drugs produced biotechnologically or by means of genetically modified organisms) have revolutionized medicine and, unlike small molecule drugs, offer significant advantages, including high specificity, potency, and safety. In particular, monoclonal antibodies (mAbs), coagulation factors, cytokines, enzyme modulators, hormones, and their analogs represent some of the most common classes of therapeutic peptides and proteins, with peptides being often even more advantageous over proteins in terms of immunogenicity, affinity, and membrane permeability. As a result, the development of peptide‐based drugs has become a vastly growing area in pharmaceutical research. The number of therapeutic peptide/protein‐based drugs has increased to more than 120 approved drugs in 2023, a trend that underscores the growing recognition of the potential of these bioactive molecules in the treatment of a wide range of health conditions [3, 4, 6, 10, 12].

Despite their success, peptides and biologics share a common challenge, which is their instability in liquid form [4]. As a result, numerous formulation techniques have been developed to enhance the stability of these therapeutic agents [13, 14]. Sources of instability can arise during both the preparation of the active agent and its storage [15]. In this context, physical and chemical characterization of peptides and proteins, as well as their formulated versions, is crucial to ensure the drug product's quality in terms of identity, purity, and activity [16, 17]. Regulatory agencies such as the US Food and Drug Administration (FDA) and the International Council for Harmonisation of Technical Requirements for Pharmaceuticals for Human Use (ICH) have established guidelines for analyzing synthesized biomolecules or biotechnological products, specifying criteria for the analysis of such therapeutics [18, 19, 20]. Nowadays, a variety of analytical techniques are available for the analysis of peptides and proteins [21]; however, they need to be adapted and validated in the course of the establishment of candidate‐specific bioanalytical workflows and monographs for testing the pure and the formulated peptide and protein drugs [22]. In particular, the aforementioned physical and chemical instability of proteins and peptides and its effect on the stability and safety of the API (active pharmaceutical ingredient) in the final product led to the establishment of guidelines that typically include recommendations concerning the following criteria: (1) characterization of the biological product, (2) stability testing, (3) bioanalytical method validation, (4) quality control, (5) clinical studies, and (6) packaging and storage [19, 20, 23, 24, 25]. These guidelines are developed by regulatory authorities such as the FDA, ICH, and European Medicines Agency (EMA) and are intended to ensure that biological products meet the required standards for public health and regulatory compliance.

### 1.1 Regulatory Guidelines for the Analysis of Biologics

**SCHEME 1:**
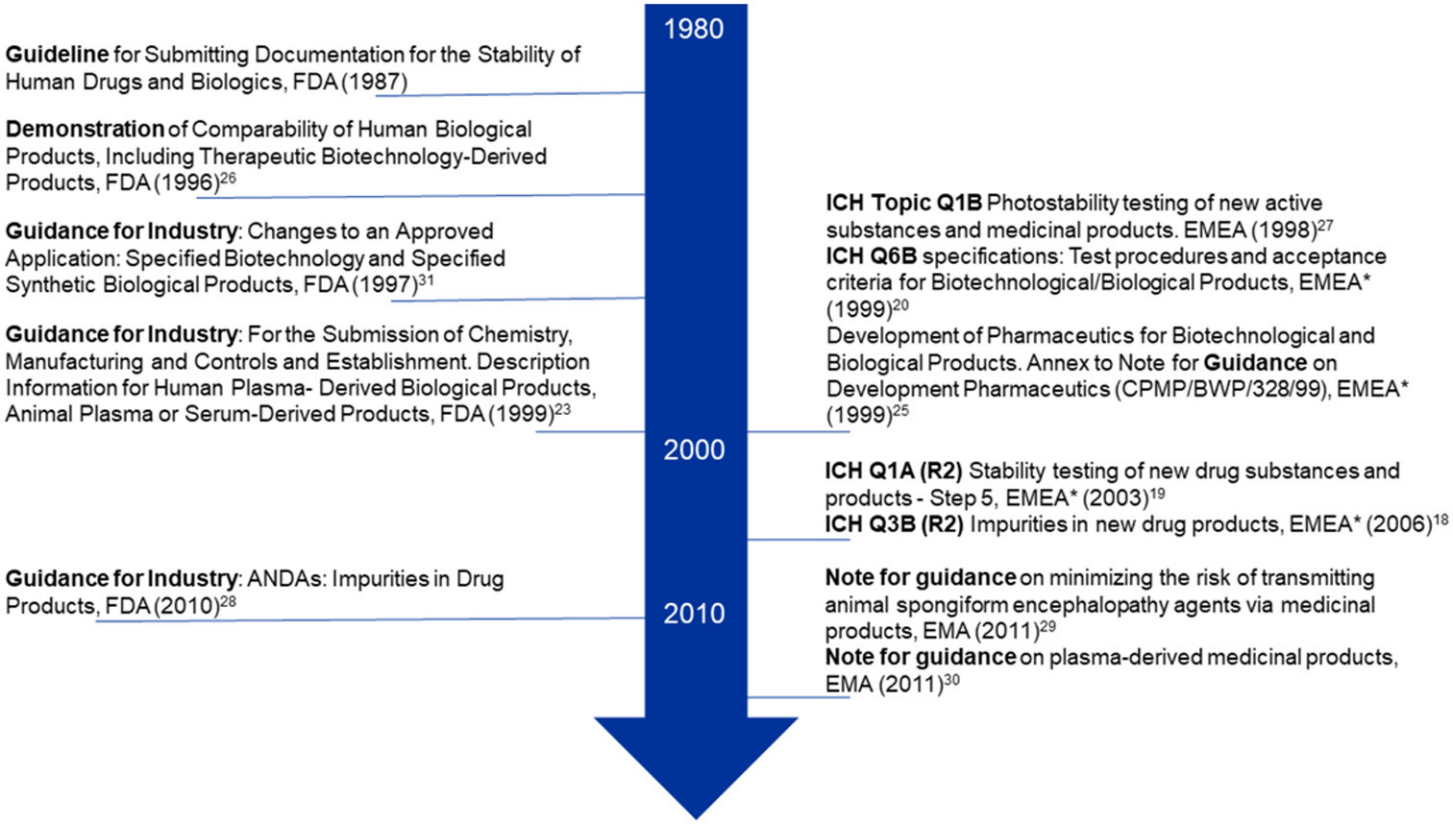
Overview of the development of regulatory guidelines provided by the FDA and the EMA since 1987 (*since 2010 EMA) [18, 19, 20, 22, 23, 25, 26, 27, 28, 29, 30, 31].

The criteria to ensure the quality, efficacy, and safety of biological products are provided and regulated by the Center for Biologics Evaluation and Research (CBER) and the Center for Drug Evaluation and Research (CDER) at the US FDA, as well as by the EMA. These guidelines are to be used as a framework for testing, specifying, and evaluating (including the conduct of clinical studies) biological products for either human or animal use that are developed as new drug entities [18, 19, 23, 24, 26, 27, 28, 29, 30, 31]. In addition, industrial guidelines exist to regulate packaging materials and control impurities in the final product. The key draft guidelines for biological products that researchers and industry professionals should follow when developing new or modified biologicals intended for human or animal use are summarized in Scheme 1.

The guidelines outlined in Scheme 1 specify the key parameters to be tested during the handling of biological products at all stages of their development, including (1) raw materials (purified peptides/proteins and qualified excipients used in the formulation), (2) manufacturing facilities (to ensure that the equipment is calibrated and the facility complies with Good Manufacturing Practice (GMP) standards), (3) analytics laboratories (for sequential and structural analysis, chromatography or electrophoresis, and bioassays), and (4) (long‐term) stability studies (utilizing stability chambers and testing under various conditions, including temperature, humidity, and light exposure). The ICH Q6B specifications [20] serve as the primary reference for evaluating biological products, setting forth essential acceptance criteria for testing and evaluation, including visual inspection, biological activity assays, immunochemical evaluation, purity testing, and quantification procedures (Table 1).

**TABLE 1:** Evaluation criteria for biological products according to ICH Q6B specifications [20].

Visual inspection	Evaluating the product's appearance and identifying the biological substance by characterizing its physicochemical properties, including structural information and post‐translational modifications (PTMs)
Biological activity assays	Analysis of the potency of the product to be evaluated
Immunogenicity/immunochemical properties	Assessing the immunochemical properties of the product (e.g., antibodies)
Purity testing	Establishing and conducting suitable tests for product‐ and process‐related impurities
Quantification procedures	Establishing methods for quantifying the peptide/protein content in relation to biological assays

Furthermore, the ICH Q1A (R2) guidelines [19] provide instructions for the stability testing of biological products, in alignment with the Q6B criteria [20]. Accelerated stability studies help to assess the shelf life and simulate long‐term storage under regular storage conditions. Stress stability conditions, including temperature, humidity, and photolysis, are designed to reflect the potential long‐term effects of storage under various conditions. These are selected based on whether the product is being tested under long‐term, intermediate, or accelerated conditions. For example, for biopharmaceuticals, it is recommended that they are to be stored at 2°C–8°C, with testing intervals adjusted accordingly, as shown in Table 2. If the drug substance is intended for storage at −20°C, only long‐term storage conditions will be evaluated at −20°C ± 5°C for a 12‐month period.

**TABLE 2:** Different conditions to be analyzed during stability studies of biological substances or products (modified from ICH Q1A [R2] guidelines [19]).

Study period	Minimum storage time	Storage conditions
Long‐term	12 months	5°C ± 3°C
Intermediate	6 months	25°C ± 2°C/60% RH ± 5% RH
Accelerated	6 months	40°C ± 2°C/75% RH ± 5% RH

Abbreviation: RH refers to relative humidity.

When the product is intended for use in a liquid form, the stability of the peptide/protein across a wide range of pH values should be thoroughly evaluated [19]. Additionally, the stability of the container and packaging materials is an essential component of the stress stability studies for the final biological product [20]. These stress tests help to identify potential degradation products, clarify the degradation pathways of each active ingredient and excipient, and validate the analytical methods to be used in the assessment [19].

Pharmacopeia specifications (monographs) are documented for each drug, including the analytical procedures for both the drug substance and the final drug product [32]. The acceptance criteria for these analytical procedures can be adjusted depending on the nature of the drug and the specifics of the analysis matrix. It is important that the described analytical methods are validated in accordance with the guidelines outlined in ICH Q2 (R2) [33], which sets the standards for the validation of analytical procedures.

### 1.2 Validation Parameters for Biological Drugs

The analysis of peptides, proteins, and biological matrices requires specific methods and precautions; thus the validation parameters for such compounds to be used as drugs have been adapted, and suitable bioanalytical methods have been introduced in ICH M10 [34]. These validation parameters are outlined and summarized in Table 3. While these parameters apply to a variety of analytical techniques, chromatographic methods and ligand binding assays are particularly favored for the quantification of biological products due to their high sensitivity and selectivity. Each analytical method should be evaluated on a case‐by‐case basis, and the level of validation required—whether full, partial, or cross‐validation—depends on the modifications made to previously validated methods and the impact these changes may have on the analytical response. In addition, the stability‐indicating properties of the analytical method are crucial and must be validated by testing the response to degradation products, as specified in the ICH guidelines. The most recent updates in quality assurance guidelines for the validation of analytical procedures are outlined in ICH Q14 [35] and incorporated into the latest drafts of ICH Q2 (R2) [33] and ICH M10 [34]. These updates ensure that the entire process of method validation and its application in the quality control of biopharmaceuticals represents the current state of knowledge and is aligned with modern standards. Advancements in instrumentation have greatly contributed to the development of various analytical techniques and the validation of bioanalytical methods for peptide or protein characterization (Table 3). When performing analytical comparisons, it is essential to consider the availability of reference standards with high purity and quality, as these are critical for guiding method evaluation.

**TABLE 3:** Parameters used for the validation of bioanalytical methods according to ICH Q2 (R2) [33] and ICH M10 [34].

Validation parameter	Definition	Conduction
Selectivity/specificity	Ability of an analytical method to differentiate and measure the analyte in the presence of potential interfering substances such as impurities, metabolites, and structurally related degradants	Observation of the response of the interfering substances in comparison to the analyte
Range	Regression model describes the range of the relationship between the nominal analyte concentration and the response of the analytical platform to the analyte (linear, nonlinear, or multivariant).	Calibration curve of at least six concentrations, including blank sample, lower limit of quantification (LLOQ), and upper limit of quantification (ULOQ), repeated three independent times on at least 2 days; signal‐to‐noise ratio can be used for calculation of detection limit and quantification limit.
Accuracy	Comparison of the measured results with expected values from the calibration curve, which represents the repeatability of the response compared with the obtained concentration.	Levels of quality controls are selected, and each is measured in at least three to five replicates in at least three runs over two or more days and depends on whether the evaluation is done for within‐run or between‐run accuracy; standard deviation from expected values is calculated.
Precision	Degree of closeness of the measured results, which represents reproducibility of the response compared with concentration, evaluates inter‐day or between‐run precision.	The same levels of quality controls are measured in at least three to five replicates in at least three runs on at least 2 days and depend on whether the evaluation is done for within‐run or between‐run precision; percent coefficient of variation (%CV) is calculated.
Carryover	Expresses the alteration of a measured concentration due to residual analyte from a preceding sample that remains in the analytical instrument.	Assessed by analyzing blank samples after the calibration standard at the ULOQ
Dilution integrity	Assessment of the sample dilution procedures	Dilution quality control is prepared by diluting a stock solution of a concentration higher than ULOQ, and at least five replicates are measured.
Robustness	Evaluation of the effects of minor changes in the analytical parameters on the response	Modifications in the analytical parameters (e.g., pH, temperature, flow rate) are introduced, and the standard deviation of the measured values compared with the expected is evaluated.
Stability	Evaluation of the impact of sample preparation, storage, and storage conditions on the quality of the analyte	Different storage conditions are tested: stability of stock and working solutions, benchtop stability, freeze–thaw stability, and long‐term stability.
Reinjection reproducibility	Evaluation of the reproducibility of analyte response in case of repeated analysis of the samples (e.g., indication for equipment failure)	Same samples are reinjected and analyzed at different concentrations measured in five replicates.

According to the aforementioned guidelines, newly developed biological products need to be evaluated by a series of validated analytical methods that should be tested and optimized initially for the pure biological substance and then applied to the respective formulated product. Emerging impurities and other ingredients during the analysis process should be removed by means and methods that do not alter the physicochemical properties of the active ingredients [22, 34, 36]. One further important aspect in the analysis process is sample preparation that needs to be performed in an appropriate way to not interfere with the requirements of the specific method applied [37]. In conclusion, specific bioanalytical workflows and protocols are to be developed according to the product and formulation to be tested and evaluated.

### 1.3 Characterization of Peptides and Proteins as Pharmaceuticals

As outlined in the introduction, the importance of therapeutic peptides and proteins has steadily grown, and their complex nature had to be considered at all stages of production, testing, and quality control, yet also for storage and handling. Accordingly, the need for proper characterization of the physicochemical properties of the therapeutic biological molecule and for monitoring their integrity and stability was raised by the authorities. Various analytical techniques can be performed and applied for peptide and protein analysis, including the identification of the structure, post‐translational modifications, charge, folding state, purity, bioactivity, and the concentration of the peptide or protein of interest [38, 39]. In addition, protein degradation products should be determined by the analytical methods applied in order to differentiate the main product from structurally related impurities that could have no, differing, or even opposite biological activity [15]. Because such impurities can also be introduced by the raw material used, both the quality of the raw material and the final product should be assessed and evaluated. Therefore, the characterization needs to be performed at the different stages of production, analysis, handling, and storage of the therapeutic peptide or protein. The analytical techniques used nowadays are classified according to the measured analytical feature by the instrument and the physical/chemical stability problem [40, 41, 42, 43, 44, 45]. Table 4 reveals an overview of the currently applied analytical techniques for characterizing peptide/protein formulations.

**TABLE 4:** Analytical methods suggested for the characterization of therapeutic peptides and proteins, including reference to the involvement in quality control (QC) routine analysis of peptide/protein pharmaceuticals (modified from [15, 16, 40, 41, 42, 43, 44, 45]).

Stability parameter	Analysis method	Result/outcome of analysis	QC
Chemical changes	RP‐HPLC	Hydrophobicity, purity, peptide/protein degradation, concentration	Yes
	LC–MS	Molecular weight	No
	IEC	Charge	Yes
	IEF	Charge, isoelectric point (pI)	Yes
	Zeta potential	Charge	No
Structural and conformational changes	DSC, DSF	Thermal parameters (e.g., melting point T_m_)	No
	ED	Primary structure, disulfide connectivity	No
	LC–MS/MS	Primary structure, disulfide connectivity	No
	CD	Secondary and tertiary structure	No
	Fluorescence	Tertiary structure	No
	UV/vis	Tertiary structure	Yes
	Raman, IR	Secondary structure, chemical characterization	No
	NMR	Secondary and tertiary structures	No
Tendency for aggregation	SDS‐PAGE	Molecular weight	Yes
	ce	Molecular weight	Yes
	AUC	Molecular weight, shape	Yes
	MS	Molecular weight, charge, aggregates	No
	SEC	Hydrodynamic size	Yes
	DLS, NTA	Hydrodynamic size	No
	Optical microscopy	Size/morphology	No
	Fluorescence microscopy	Size, morphology, aggregates	No
Activity assay	ELISA (visible)	Specific binding sites/concentration	Yes
	EPR	Binding to ligand	No
	SPR	Binding to ligand	No

Abbreviations: AUC: analytical ultracentrifugation, CD: circular dichroism, ce: capillary electrophoresis, DLS: dynamic light scattering, DSC: differential calorimetry, DSF: differential scanning fluorimetry, ED: Edman degradation, ELISA: enzyme‐linked immunosorbent assay, EPR: electron paramagnetic resonance spectroscopy, IEC: ion exchange chromatography, IEF: isoelectric focusing, IR: infrared spectroscopy, LC: liquid chromatography, MS: mass spectrometry, NMR: nuclear magnetic resonance spectroscopy, NTA: nanoparticle tracking analysis, RP‐HPLC: reversed‐phase high‐performance liquid chromatography, SDS‐PAGE: sodium dodecyl sulfate‐polyacrylamide gel electrophoresis, SEC: size‐exclusion chromatography, SPR: surface plasmon resonance spectroscopy, UV/vis: ultraviolet–visible spectroscopy.

Changes in the thermodynamic properties of peptides and proteins can be assessed using techniques such as differential scanning calorimetry (DSC) and differential scanning fluorimetry (DSF) [46], which measure the melting temperature (Tm) upon unfolding of the protein. This temperature reflects the stability of the protein's structure after formulation procedures [47]. Additionally, structural information about the peptide/protein primary structure can be obtained through tandem mass spectrometry (MS/MS) [48] and Edman degradation (ED) [49]. These methods are used to confirm mutation points and post‐translational modifications, such as disulfide bridges or glycosylation sites within the amino acid sequence [50, 51, 52]. Peptide mapping or sequencing can also be valuable for identifying potential chemical degradation or isomerization instability. To assess secondary and tertiary protein structures, various spectroscopic methods are employed, including circular dichroism (CD) [53, 54], fluorescence and Fourier transform infrared (FTIR) spectroscopy [55], X‐ray crystallography [56], Raman spectroscopy, and nuclear magnetic resonance spectroscopy (NMR) [57]. Application of these methods allows the detection of unfolding, degradation, aggregation, or hydrolysis of the native peptide or protein and thus evaluation of the compound's chemical and physical stability. Chromatographic techniques play a central role in peptide/protein analysis, particularly based on the protein's hydrophobicity, mass, and charge. For example, reversed‐phase high‐performance liquid chromatography (RP‐HPLC) is commonly used to assess signs of degradation and to separate and quantify the active therapeutic protein from impurities or degradants [58, 59]. Other techniques, such as size exclusion chromatography and ion‐exchange chromatography, are also widely used to evaluate protein quality. These methods are often coupled with various detectors, such as UV, fluorescence, or refractive index detectors, and have been successfully applied to numerous proteins and peptides [60]. As a result, chromatography‐based techniques not only provide qualitative insights into protein stability but also enable quantitative assessments of stability and purity [61, 62, 63, 64, 65].

Electrophoresis methods, such as sodium dodecyl sulfate‐polyacrylamide gel electrophoresis (SDS‐PAGE) [66], native PAGE [67], isoelectric focusing (IEF) [68], and capillary electrophoresis (ce) [69, 70, 71], are commonly used for the quality control of proteins and, due to the sequence length and technical issues, to a much lesser extent of peptides. These methods take advantage of the protein's charged ionization properties and its behavior in an ionic medium [60]. Differentiation of degradation products or protein aggregates from the native molecule can be achieved based on changes in their charge and/or size. For further evaluation, light scattering techniques can be applied, such as dynamic light scattering (DLS) [72, 73] and nanoparticle tracking analysis (NTA) [74], to measure the particle size and/or molecular weight of unstable protein molecules by detecting light intensities as a turbidity indicator.

To evaluate the functional activity of a protein or peptide, specific analyses are essential [72]. This includes identifying ligand interactions and testing binding upon administration of the therapeutic biological drug. In vitro or in vivo testing is performed, typically using distinct methods to assess the binding affinity and capacity of the peptide or protein to its specific receptor or ligand [75]. Techniques such as microscale thermophoresis (MST), isothermal calorimetry (ITC), and the current gold standard, surface plasmon resonance (SPR) spectroscopy [76, 77], can be used to analyze ligand binding by monitoring the interaction between the ligand and the receptor molecule that is, in the latter case, immobilized on an inert gold surface. Thereby, the reflection of light from the metal surface at a specific angle corresponds to the mass increase resulting from ligand–protein interactions [75]. Furthermore, enzyme‐linked immunosorbent assays (ELISAs) are widely used to test the binding of proteins to specific antibodies. Binding site specificity is crucial in the design of ELISA assays, which involve antibody‐coated plates and enzyme‐labeled antibodies to enable detection [78, 79]. The most commonly used enzyme labels in ELISA are horseradish peroxidase (HRP) or alkaline phosphatase (AP). These enzymes catalyze the oxidation of a substrate into a colored product, which is then measured spectrophotometrically to quantify the binding interaction [15, 80, 81]. Apart from binding analysis, special active ingredient‐dependent functional analyses are carried out, which can range from, e.g., spectrophotometric enzyme activity assays to biological cell‐based functional assays.

By employing these various analytical techniques, a comprehensive characterization of the peptide or protein can be achieved, particularly for newly explored biomolecules, ensuring their identity, purity, stability, and functionality throughout the development process [39].

### 1.4 Stability Issues of Peptides and Proteins and Consequences for Their Pharmaceutical Application

**FIGURE 2:**
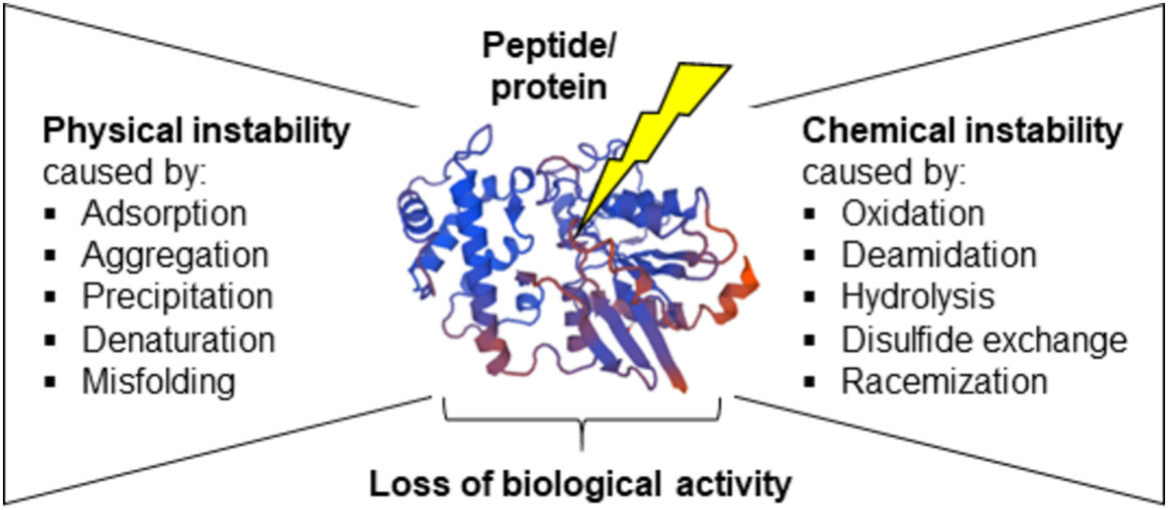
Physical and chemical changes affecting the structural integrity of peptides and proteins (modified from [82, 83, 84]).

The challenge of peptide and protein instability inevitably results from their highly complex 3D structure, with a plethora of conformations occurring in peptides owing to their high flexibility, whereas a sophisticated interplay of secondary structure elements and superordinate interactions leads to defined tertiary and quaternary structures in proteins. Various factors can lead to physical or chemical changes in the structure of proteins and peptides, such as alterations in temperature, pH, ionic strength, or interfacial exposure between the biological molecule and the surrounding medium [82]. Different stages of the lifecycle, including production, purification, transfer, and storage, may cause these changes, leading to different types of instability that can affect the biological activity of a peptide or protein as outlined in Figure 2.

Physically, exposure to these factors can cause the protein to lose its native folding, undergo degradation, or form aggregates. Protein aggregation can be either reversible or irreversible. Irreversible aggregation, particularly non‐native aggregation, leads to the denaturation of the protein, which can result in the formation of, e.g., fibrils. These aggregates can trigger undesirable immunological responses within the human body, posing significant challenges for the development of therapeutic proteins and peptides [85, 86, 87]. Protein aggregation can arise from both physical and chemical degradation processes (Figure 2). Chemical degradation‐driven aggregation occurs through various modifications to the protein structure, such as oxidation, deamidation, hydrolysis, and disulfide bond shuffling [40, 87]. For instance, oxidation of amino acid residues in the peptide/protein chain may occur when proteins are exposed to light, oxidizing agents (e.g., oxygen, peroxides), free radicals, or metal ions. Methionine and cysteine are the amino acids most prone to oxidation, though histidine, tyrosine, tryptophan, and phenylalanine can also be affected [88, 89]. The likelihood of oxidation depends on the location of the amino acids: core amino acids are less exposed and therefore less prone to oxidation than terminal amino acids. For example, methionine residues in globulins and unpaired cysteine residues are particularly vulnerable to oxidation and degradation [90]. Free cysteines can undergo oxidation, forming disulfide bonds or engaging in thiol‐disulfide exchange reactions, which can ultimately lead to protein aggregation. A well‐known example of such aggregation occurs in the basic fibroblast growth factor (bFGF), a potent polypeptide indicated for wound healing and enhancing cellular functional recovery in myocardial infarction and bone fractures as demonstrated in studies by Wang et al. [81, 91, 92]. Additionally, the rate of oxidation reactions increases with higher pH levels in the surrounding environment, and disulfide scrambling can occur even in the absence of free cysteine residues. The cleavage of disulfide bridges and/or isomerization are common degradation processes and are considered the most prevalent pathways for chemically induced aggregation (Figure 2) [93, 94], largely due to the crucial role that disulfide bridges play in peptide and protein folding [95]. On the other hand, deamidation of amino acids, which involves the loss of ammonia, alters the molecule's properties, including its hydrophobicity, charge, and mass. Deamidation of asparagine and glutamine has been observed at neutral or alkaline pH [90, 96, 97, 98]. Additionally, amino acid combinations such as asparagine‐glycine, asparagine‐serine, and asparagine‐threonine have been found to be particularly prone to deamidation, whereas glutamine is less susceptible [40].

Protein hydrolysis is a severe form of degradation that occurs under highly acidic or basic conditions. Asparagine is particularly vulnerable to hydrolysis within the protein sequence [40]. Although protein hydrolysis is not expected under typical preparation or storage conditions, it should still be considered when evaluating the conditions under which the protein is handled. Additionally, proteins are prone to adsorption onto surfaces, which can lead to reduced availability and denaturation. The selection of appropriate containers is as important as choosing the right solvents for the bioactive agent. Table 5 summarizes potential causes and sites of protein denaturation, highlighting the importance of careful monitoring of conditions and the duration of protein exposure. Numerous stress factors can arise during protein handling, storage, shipping, transfer, and manufacturing [16]. With increased understanding of protein instability—particularly in liquid form—researchers have focused on developing protective techniques to improve protein handling and preserve their integrity and desired bioactivity.

**TABLE 5:** Stability issues observed for proteins, including their possible causes and sites of contact with stressful conditions (modified from [16]).

Stability issue	Causes	Condition/occurrence	Solutions
Non‐covalent aggregation (precipitation or aggregation)	Structural changes, solubility, mechanical shear, impurities, heat, humidity	Storage, shipping, handling, delivery, container integrity, lyophilization, accidental freezing	Temperature control, purity of raw material, pH optimization, ionic additives
Covalent aggregation	Disulfide scrambling	pH changes at any stage of handling	pH optimization, protein denaturants
Deamidation	pH < 5.0 or > 6.0	pH changes at any stage of handling	pH optimization
Cleavage and hydrolysis	Highly acidic or alkaline pH, protease impurities	pH changes, impurities	pH optimization, product purity
Oxidation	Active oxygen species, free radicals, metal impurities, light	Light exposure during storage, excipient instability, or impurity	Free radical scavengers, active oxygen scavengers
Surface denaturation	Protein hydrophobicity, specific affinity to surfaces	Container incompatibility, impurities	Surfactants, container material selection

In summary, both physical and chemical factors contribute to the instability of peptides and proteins, and these processes can significantly affect their quality, efficacy, and safety in pharmaceutical applications. Understanding these degradation pathways is crucial for developing effective strategies to stabilize protein‐ and peptide‐based therapeutics.

### 1.5 Stabilization of Pharmaceutical Peptides and Proteins

Given the inherent nature of peptides and proteins and their susceptibility to degradation (denaturation) as discussed earlier, pharmaceutical scientists have applied their expertise to develop various strategies and delivery systems to meet the needs of functional therapeutic agents for proper administration [83]. Because protein therapeutics primarily target extracellular sites, their effectiveness often depends on factors such as sequence length and amino acid composition [99]. However, for precise control of distinct medical conditions, intracellular delivery is highly desirable. This has led to an increasing demand for the design of more efficient therapeutic agents. A significant advancement in the development of therapeutic proteins and peptides has been the introduction of sequence modifications and engineering (either chemical or biological) aimed at improving the pharmacological properties, such as enhancing potency, delivery/permeability, reducing side effects, and improving stability/half‐life [11]. The strategies used to engineer peptides and proteins can be categorized into genetic and biotechnological engineering, as well as covalent and non‐covalent modification approaches [15]. Genetic engineering involves techniques such as site‐specific mutagenesis and the development of more stable amino acid analogs with desirable pharmacokinetic properties. For example, human recombinant insulin has been modified through a range of genetically substituted amino acid analogs, each offering varying degrees of stability and activity and enabling personalized therapy for diabetes patients [100, 101, 102].

Covalent fusion of proteins with other molecules is an effective strategy in protein engineering using, for example, Fc‐fusion, i.e., the Fc region of an antibody is fused to the protein of interest, significantly increasing its stability and half‐life. A well‐known Fc‐fused protein is etanercept, used in the treatment of rheumatic diseases and psoriasis. This protein, derived from the extracellular domain of the p75 tumor necrosis factor receptor (TNFR) fused with the Fc portion of an IgG antibody, exhibits a longer half‐life in the bloodstream compared with its unfused counterpart. This fusion increases the molecular size, promoting endosomal recycling and extending the duration of action [103, 104]. Similar improvements in stability and activity have been observed with the covalent attachment of polymers, sugars, or lipids to proteins. The covalent attachment typically targets functional groups on the protein's N‐ or C‐termini, thiol groups, or exposed tyrosine and lysine residues that can undergo chemical modification. Importantly, these modifications must not interfere with the protein's active site or its receptor‐binding interface to preserve therapeutic efficacy [105].

PEGylation, the conjugation of proteins with polyethylene glycol (PEG), is another prominent example of covalent modification. More than 40 FDA‐approved protein drugs have been PEGylated, enhancing their stability, particularly in the digestive system [15]. Moreover, conjugation to human serum albumin (HSA) was shown to enhance the bioavailability and increase the half‐life of small molecule drugs dramatically [106], and thus was considered in peptide/protein drug development, too. Similarly, lipidation of the peptide at distinct amino acid side chains, e.g., lysine, can significantly improve stability and bioavailability. Notable examples of lipidated peptides include liraglutide [107] and insulin detemir [108], both of which have demonstrated increased stability and therapeutic effectiveness. Other modifications, particularly for peptides, include cyclization, the insertion of unnatural amino acids [11], disulfide mimetics, and stapling via intrachain bridges [7, 109, 110] to enhance stability. Additionally, glycosylation, the attachment of sugar molecules to proteins, can improve stability, solubility, and activity, and enable the formation of additional hydrogen bonds with hydrophilic residues on the polypeptide backbone, enhancing the protein's overall properties. For instance, a glycosylated p‐succinamidophenylgluco‐pyranoside‐insulin conjugate (SAPG‐insulin) has been shown to exhibit a reduced tendency to form dimers or fibrils [111].

Non‐covalent engineering of therapeutic proteins and peptides primarily occurs through formulation, a process that is extensively studied to maintain the integrity of the therapeutic agent throughout storage and handling until it is administered to the patient [112]. The selection of excipients is tailored to the specific characteristics of the drug molecule being delivered. As such, solvents, excipients, and formulation methods must be carefully chosen to achieve and/or preserve the desired pharmacological and pharmaceutical properties [84, 113].

Compared with conventional small‐molecule pharmaceuticals, developing a stable protein formulation requires significantly more resources and effort due to the complex and delicate nature of proteins and their structural stability [114]. The pre‐formulation process begins with obtaining the protein in its native form at the highest possible purity. It is crucial to investigate the structural, physical, and biological properties of the target molecule. In addition to understanding its structural characteristics (primary, secondary, tertiary, and quaternary structures), the physicochemical properties of the therapeutic protein, such as molecular weight, solubility, extinction coefficient, stability, aggregation propensity, and native post‐translational modifications (e.g., glycosylation), must also be thoroughly examined [16, 83]. Furthermore, the biological activity, substrate or receptor affinity (through in vivo or in vitro models), and pharmacokinetic profile of the therapeutic molecule must be assessed before formulation [83]. Given that the therapeutic efficacy of proteins and peptides is largely dependent on their conformational structure, these preliminary data are essential for designing and developing a formulation that preserves the protein's structural integrity in its native form. Pre‐purification steps, such as precipitation or separation from a biological mixture, may be necessary before formulation, depending on the quality of the supplied protein [82]. Additionally, selecting appropriate excipients is a critical step in the formulation process [84]. Qualified excipients must be thoroughly characterized, including detailed information about their origin, structure, physicochemical properties, function, stability, and compatibility with both other excipients and the active ingredients [84, 115, 116]. The intended indication and route of administration will influence the choice of excipients, as well as their potential for stabilizing the pharmaceutical product. Various classes of excipients, outlined in Table 6, are categorized based on their effectiveness and stabilizing properties [84].

**TABLE 6:** Excipients currently used in formulations of pharmaceutical peptides or proteins (modified from [84]).

Excipient		Aimed formulation effect
Type	Example	
Surfactant	Poloxamer [117] Polysorbate 20 and 80 [118]	Anti‐adsorption, cryoprotectants, lyoprotectants
Polymers	Dextran [119] Cyclodextrin [120, 121] Polyethylene glycol (PEG) [122, 123, 124] PVP [122, 123, 125] PLGA [126, 127, 128]	Anti‐adsorption, stabilizers
Sugars	Glucose [129] Sucrose [122, 123, 125, 127, 129] Trehalose [122, 123, 130, 131]	Stabilizers, cryoprotectants, lyoprotectants
Polyols	Glycerol [122, 123, 132] Mannitol [122, 123, 133] Sorbitol [116, 122, 123, 132]	Stabilizers, cryoprotectants, lyoprotectants
Antioxidants	Ascorbic acid [122, 134] Ectoine [116, 135] Glutathione [122] Monothioglycerol [136] Vitamin E [40, 137]	Oxidation protection
Chelating agents	Citric acid [40, 137] EDTA [134, 138] Thioglycolic acid [40, 137]	Oxidation protection
Buffer salts	Phosphate, bicarbonate, sulfate, nitrate, acetate, chloride, pyruvate [137, 139]	pH control, stabilizer, tonicity modifier
Antacids	Mg(OH)_2_ [127] ZnCO_3_ [128]	pH control
Amino acids	Alanine [122, 123], arginine [122, 128, 140] Aspartic acid [122], glycine [122, 123] Histidine [141], lysine [122] Proline [122, 123, 142]	Stabilizer, solubilizer
Ligands	Phenol [143] Zinc [144]	Stabilizer
Others	HSA, BSA [145]	Stabilizer, anti‐adsorption

Abbreviations: BSA: bovine serum albumin; EDTA: ethylenediamine tetra acetic acid; HSA: human serum albumin; PEG: polyethylene glycol; PLGA: poly(lactic‐co‐glycolic‐acid); PVP: poly(vinyl pyrrolidone).

Due to the nature of proteins and peptides, they are usually prepared in a lyophilized form ready for reconstitution prior to administration, mainly as an injectable dosage form (intramuscular, intravenous, or subcutaneous) and under refrigerated storage (2°C–8°C). Incorporation of numerous excipients, such as polymers, sugars, and cyclodextrins, facilitates the injection‐free administration, i.e., transdermal, pulmonary, ocular, nasal, rectal, and vaginal routes [146, 147, 148, 149]. Moreover, the oral administration of peptides and proteins gained an increasing interest over the years in favor of patient convenience and compliance. Gastrointestinal tract stability is a critical concern for the technologists. Therefore, many approaches were optimized for protecting and stabilizing the administered protein/peptide and, at the same time, ensuring their biological activity. This includes, namely, cyclization, PEGylation, attachment to cell‐penetrating peptides, and prodrug formation [61, 62, 63, 64, 65, 150]. Particulate formulation systems were developed as a carrier system for proteins and peptides, such as emulsions, micro‐ and nanoparticles, solid core particles, and liposomes, and they have successfully modulated the release rate and targeting of the formulated protein, in addition to the possibility of oral delivery [150, 151, 152]. Specific medical devices were fabricated for controlled, targeted release of therapeutic proteins and peptides, such as biodegradable microneedle‐based delivery systems [153], ingestible self‐orienting systems [154], and intestinal mucoadhesive patches [155].

The formulation of proteins for any delivery route begins with the handling of raw materials, followed by numerous processing steps that may also lead to protein or peptide denaturation [114]. Therefore, the environment in which proteins are handled during formulation needs to be adjusted to stabilize the formulated active ingredient, and factors affecting protein stability must be closely controlled [89]. Among these factors that may contribute to process‐related degradation are the purity of the solvents and excipients used, moisture content, the pH of the formulation, sterility if required, and uncontrolled temperature conditions. Additionally, water plays a critical role in protein degradation, so solid protein formulations are commonly prepared by removing water through evaporation and atomization (e.g., spray drying) or sublimation (e.g., freeze drying or spray freeze drying) of liquid protein solutions [89, 156, 157].

When proteins are exposed to atomization, dehydration, freezing, interfacial, and/or shear stress during the drying process, water removal can increase the likelihood of process‐related denaturation through, e.g., aggregation, unfolding, oxidation, and/or deamination [158]. Ultimately, this can result in the loss of the protein's structure and functionality [65, 89, 159]. Another mechanism for protein stabilization in its solid form is the formation of an amorphous glass matrix composed of polyols, sugars, and polymers, in which protein molecules are immobilized and embedded [160]. This technique forms the basis of the so‐called hot‐melt extrusion (HME), a novel stabilization method for embedding drug molecules that significantly reduces protein mobility and the tendency for degradation, unfolding, or interaction with surrounding media, thereby increasing the stability of the extruded protein or peptide [157, 161].

**FIGURE 3:**
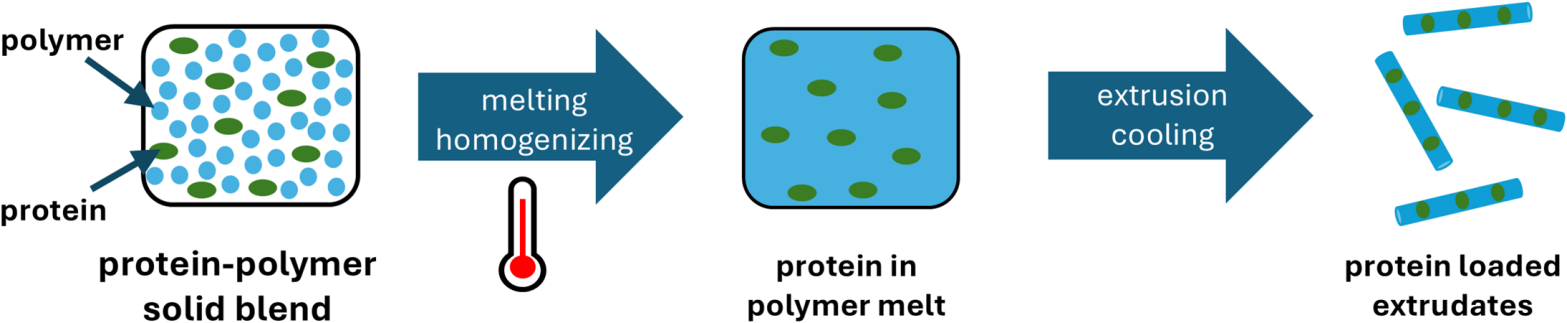
Schematic representation of the hot‐melt extrusion (HME) procedure applied to peptides and proteins to be stabilized (inspired from [162, 169]).

HME of a protein begins with filling a preheated ram extruder with a physical mixture of powdered protein and polymer, which is then blended and melted in a cylindrical barrel. The molten mixture is subsequently pressurized and forced through a die, where it is transformed into rods of the desired size, as shown in Figure 3 [162].

In general, HME offers several advantages for formulating proteins into solid dosage forms with a solvent‐free technique, eliminating the need for water or organic solvent removal. It also allows for the handling of highly potent proteins in a closed system with fewer excipients and has potential for easier scale‐up to production levels [163]. Additional benefits include taste masking, improved bioavailability, and controlled and targeted release of the biologic drug from the matrix, making HME an attractive formulation technology for protein delivery systems [162, 164, 165]. However, due to the heated system, the formulation components must be thermally stable at the melting and extrusion temperatures. The large batch sizes required for drug substances and excipients also increase the development costs [166, 167, 168]. The comprehensive use of the method must be tested on numerous peptides and proteins in the future in order to be able to derive general statements about its applicability. Proper analytical characterization of the treated protein will provide a better understanding of the stability of the active ingredient. In general, all advancements in protein delivery systems rely on the availability of the biological drug molecules in their active state. Thus, validated methods and workflows for monitoring the physicochemical properties of the active peptide or protein are required throughout all processes it undergoes before entering the market.

## 2 Conclusions

The development and application of peptides and proteins as therapeutic agents require strict adherence to guidelines and standards issued by organizations such as the FDA, EMA, and ICH. These guidelines ensure the quality, efficacy, and safety of these biological products at every stage of the production process, starting from raw material sourcing to testing the final product stability. Detailed testing protocols define manufacturing, analytical evaluation, and stability studies, including stress tests under various conditions to simulate long‐term storage and ensure product integrity. The ICH Q6B guidelines, in particular, should be taken into account as a key reference for the evaluation of peptide and protein drugs with regard to parameters such as biological activity, purity, and stability, with the latter being integral to determining shelf life and assessing potential degradation pathways. Additionally, pharmacopeia specifications and validated analytical methods play a vital role in the characterization and quality control of therapeutic biological products.

The analytical techniques for the validation and characterization of peptides and proteins are highly specialized, requiring methods like chromatography, mass spectrometry, and electrophoresis that allow for the detection of impurities, degradation products, and conformational changes and ensure that the final product meets the required specifications. Functional assays, such as ligand binding and ELISA, assess the biological activity of the therapeutic products, which is crucial for their efficacy. Furthermore, peptides and proteins are inherently unstable due to their complex three‐dimensional structures, which make them susceptible to both physical and chemical degradation. Factors like temperature, pH, ionic strength, and interfacial exposure can alter their native conformation, leading to denaturation, aggregation, or degradation. Such instability can significantly affect their biological activity and, in turn, their therapeutic efficacy. Aggregation, particularly irreversible non‐native aggregation, is a major challenge for protein stability, as it may lead to the formation of immunogenic fibrils. Chemical degradation processes like oxidation, deamidation, hydrolysis, and disulfide bond shuffling further exacerbate the problem. These chemical changes, especially in key residues such as methionine and cysteine, can disrupt protein folding and cause aggregation, while processes like deamidation alter the protein's properties, impacting its stability and bioactivity.

Despite these challenges, various stabilization techniques have been developed to preserve the integrity and bioactivity of therapeutic peptides and proteins. One approach involves genetic and biotechnological engineering, which allows for modifications to enhance the stability and pharmacokinetic properties of these biomolecules. Covalent modifications like PEGylation, lipidation, and Fc‐fusion can significantly improve the stability, half‐life, and delivery efficiency of therapeutic proteins. These modifications are designed to prevent denaturation and aggregation while ensuring the protein maintains its functional properties, such as receptor binding and enzymatic activity. Additionally, non‐covalent strategies such as careful formulation design and the use of excipients help stabilize proteins during storage, transportation, and administration. Finally, the formulation of peptide and protein drugs requires a deep understanding of their structural, physicochemical, and biological properties. Pre‐formulation steps are crucial to ensure the protein is in its native, functional form before it undergoes a stabilization process. In recent years, solid‐state formulations, such as lyophilized powders and HME, have provided effective strategies for stabilizing proteins during storage and delivery. HME, as an example, can embed proteins in an amorphous matrix, reducing their mobility and susceptibility to degradation. Despite the promise of such techniques, challenges remain in preserving protein stability during manufacturing, and the formulation must be carefully optimized to avoid process‐induced degradation.

In summary, the regulatory framework for biological product development focuses on ensuring that each step is thoroughly tested for quality and consistency. Advances in analytical techniques and the implementation of stringent validation processes are essential for maintaining high standards in biopharmaceutical manufacturing, ultimately ensuring the safety and effectiveness of new biological therapeutics.

## Author Contributions

All authors contributed to and approved the final manuscript.

## Conflicts of Interest

The authors declare no conflicts of interest.

## Data Availability

Data sharing is not applicable to this article as no new data were created or analyzed in this study.
